# Supplementary material for: The genome polishing tool POLCA makes fast and accurate corrections in genome assemblies
Source: PLoS Comput Biol. 2020 Jun 26;16(6):e1007981. doi: 10.1371/journal.pcbi.1007981 (PMC7347232; doi:10.1371/journal.pcbi.1007981)
Supplement: Attachment — Submitted filename: Response to reviews.docx [file pcbi.1007981.s001.docx]

Response to the reviews:

We thank the referees for their thoughtful comments that helped us improve the manuscript. Below we detail the modifications to the manuscript that we made in response to the comments. Since the submission of the manuscript in December 2019, we made significant improvements to the POLCA software, resulting in significantly improved performance and speed, making it competitive with the best polishing software available in the field. We have added a mention of the newer ntEdit, as suggested by reviewer #2, but we did not add it to our comparisons because ntEdit performed very poorly on our benchmarks, and its addition to the analysis would make our figures hard to read. We did add the comparisons to the NextPolish software, published online in *Bioinformatics* on November 28, 2019, just a short time before the POLCA paper was submitted. NextPolish turns out to perform similarly to the latest version of POLCA, and much better than Pilon and Racon on all our benchmarks.

Below are responses to the specific comments of both reviewers. Our responses are in italics to distinguish them from the reviewers’ comments.

Reviewer #1: This manuscript describes a new pipeline for correcting long-read genome assemblies using short-read sequences. POLCA was compared against Pilon and Racon and demonstrated advantages over both. Comments below are provided to improve the utility of the manuscript.

I attempted to install MaSuRCA-v3.3.5 on a local workstation running openSUSE 15.0 but received a fatal error regarding the lack of the file 'xlocale.h'. Apparently this file was removed since glibc 2.26 so I had to form a symbolic link to '/etc/local.h'. The authors should fix this in the next distribution, or at least provide this information in the Github site. It seems that someone else already reported installation errors (issues #148 and #151 in Github). Lack of clean installs will limit the use of this tool.

*Response: Thank you for bringing this system compatibility issue to our attention. We have updated the README.md document on Github, under “Installation instructions,” with directions to resolve the issue.*

The use of Freebayes for correction, and then batching for parallel correction, is very much like the pipeline described in the Vertebrate Genome Project (<https://github.com/VGP/vgp-assembly/tree/master/pipeline/freebayes-polish>). Are the authors affiliated with VGP? If so, then please credit this github repository. If not, please explain how POLCA is different from the VGP pipeline.

*Response: We are not affiliated with VGP. Although we both use FreeBayes, the POLCA pipeline is different from VGP in that it uses different FreeBayes parameters and it adds custom code for post-processing of the FreeBayes calls (instead of using bcftools, as the VGP does), which results in more accurate corrections.*

In the first paragraph of Introduction, please provide a reference to support the overall error rate of <1/100000 bases in hybrid assemblies.

*Response: the reference is to Wick et al. (2017), the paper on bacterial assemblies from hybrid data, whose results we use for our “bacterial genomes” evaluation. We added the reference at the end of the sentence in the introduction.*

On page 6 in the first paragraph of Simulated data experiments, the authors describe how the errors were introduced in the simulated Illumina reads (wgsim) - please describe how random errors were introduced in the clean genome so that one may reproduce the experiment.

*Response: the code to add random errors to an assembly is distributed with MaSuRCA. We have added the description of the commands required to the POLCA section of the README.md file on github, and we now refer to that README.md file in the revised text.*

When BWA maps an Illumina read from a repetitive region, doesn't it map the read to one of the repeats that is chosen at random, therefore isn't it possible for this read to provide incorrect correction at repetitive regions? This is an important drawback of Illumina correction of large genome assemblies so it would be useful to know how POLCA take this into account.

*Response: the ability to map Illumina reads to the right location of the genome is a limitation of all polishing algorithms, and POLCA cannot do anything about that limitation, which is inherent to short reads. It may be possible to improve mapping accuracy by using linked Illumina reads, such as the ones produced by 10x Genomics chromium platform, which would improve the polishing quality.*

On page 10, Table 3: To help the table 'stand alone', it would be useful to know the original number of errors in the genome (perhaps in the Table header) and a column at the beginning to show the number of errors after a single run of each algorithm. The reader could more easily compare the utility and advantage of a second round of polishing.

*Response: we added the total number of errors introduced to the Table caption, and we also added the “Single run” column that shows the number of errors after a single run of each algorithm.*

Minor corrections
On page 6 in the first paragraph, the authors probably want to change '00.1%' to '0.1%'.
*Done*
In the third line on page 7, 'smaller' instead of 'small'.
*Done*

Reviewer #2: The authors present POLCA, a new genome polishing tool that used Illumina data to improve genome assemblies performed using long read technologies such as Nanopore and PacBio. They state that their newly developed tool performs faster than RACON and Pilon, the two most widely used tools, and with a comparable or higher accuracy. The authors mention three main advantages of POLCA: its speed, its low memory usage and its accuracy. To demonstrate this, they use three different examples: simulated data, human data and bacterial data. Finally, they assess the effect of using a combination of strategies.

The authors performed several tests to demonstrate their claims and thus the usefulness of their tool. The manuscript is well written and easy to follow. Also, their tool is easy to install and use. While POLCA is distributed as part of the MaSuRCA package it can also be used independently, thus it is a flexible tool. Additionally, the fact that it is distributed along with a widely used genome assembler will allow it reaching a large number of potential users.

Major Points:
While the authors have compared their tool with the most widely used tools, there are some more recent tools such as NextPolish or ntEdit which also claim to be faster than RACON and Pilon and similar accuracy levels. I consider that the manuscript would benefit from comparing the performance of some of these new tools with POLCA.

*Response: we added comparisons to the NextPolish software to the manuscript. We also made improvements to POLCA, so that it now outperforms PILON and Racon by a larger margin. In comparison with NextPolish, POLCA performs very similarly, winning on some measures and losing on others. We also ran ntEdit, but its performance was much worse than the other tools, so we left it out of the tables and figures, although we reference it in the introduction.*

Minor Points:
The authors mention that “Whole-genome assemblies assembled using a hybrid sequence strategy can thereby obtain an overall error rate of less than 1 error per 100 thousand bases.” How have they calculated this number, or which is the reference used for this calculation?

*Response: as stated in our response to reviewer #1, this statement is based on Wick et al. (2017).*

The authors state that they empirically calculated the allele frequency threshold used by POLCA to be 2. I think that the manuscript would benefit from a more comprehensive description of the information and methodology used to determine the threshold.

*Response: since the submission of the paper, we modified the criteria for making a correction, based on many additional experiments on real and faux data sets. We list the new criteria in the paper, which is AO>=2*RO and AO>1, which works for wide range of coverage of Illumina reads. AO is the number of observations (count) of the alternative allele, the one different from the consensus, and RO is the number of observations of the reference allele.*
